# Interleukin-21 Accelerates Thymic Recovery from Glucocorticoïd-Induced Atrophy

**DOI:** 10.1371/journal.pone.0072801

**Published:** 2013-09-02

**Authors:** Moutih Rafei, Maude Dumont-Lagacé, Alexandre Rouette, Claude Perreault

**Affiliations:** 1 Department of Pharmacology, Université de Montréal, Montreal, Quebec, Canada; 2 Institute for Research in Immunology and Cancer, Université de Montréal, Montreal, Quebec, Canada; 3 Department of Medicine, Université de Montréal, Montreal, Quebec, Canada; University of Alberta, Canada

## Abstract

Both physiological and psychological stress cause thymic atrophy via glucocorticoïd (GC)-dependent apoptosis of double-positive (DP) thymocytes. Given the pervasiveness of stress, GC-induced thymic atrophy is arguably the most common type of acquired immunodeficiency. We recently reported that interleukin-21 (IL-21) has a unique ability to expand the small subset of DP thymocytes (CD69^+^) which are ongoing positive selection, and that administration of IL-21 increases thymic output in aged mice. The goal of this study was to evaluate whether IL-21 could mitigate GC-induced thymic atrophy. In contrast to double-negative (DN) and single-positive (SP) thymocytes, most DP thymocytes (CD69^−^) do not constitutively express the IL-21 receptor (IL-21R). Accordingly, CD69^−^ DP thymocytes from PBS-treated mice were unresponsive to IL-21 administration. However, following GC injection, surviving CD69^−^ DP thymocytes up-regulated IL-21R and responded to IL-21 treatment as evidenced by enhancement of Bcl6 expression and phosphorylation of STAT1, STAT3 and STAT5. Consequently, IL-21 administration to GC-treated mice accelerated thymic recovery by expanding considerably DP thymocytes and, to a lesser extent, DN thymocytes. However, IL-21-induced expansion of DN/DP thymocytes did not alter the diversity of the intrathymic or peripheral T-cell receptor (TCR) repertoire. We conclude that IL-21 dramatically accelerates recovery from GC-induced thymic atrophy.

## Introduction

The thymus is critical for sustained T-cell development in vertebrates [1], [2]. Nonetheless, the thymus undergoes early age-related involution characterized by a decline in thymic cellularity and output [3], [4]. Age-related thymic involution is multifactorial and hinges on two key factors: defects affecting pre-thymic hematopoietic progenitors and the loss of thymic epithelial cells (TECs) [4], [5]. In addition, thymopoiesis is exquisitely sensitive to stress. Indeed, the stress response is characterized by the triad of enlarged adrenal glands, gastric erosions and thymic atrophy [6]. GCs produced by hyperactive adrenal glands trigger apoptosis of DP thymocytes in an Apaf-1- and caspase-9-dependent manner [4], [7]. Common stressors include bacterial/viral infections [8]–[11], starvation/malnutrition [10], [12] and psychological stress [12]–[14]. GCs are also widely used in the treatment of autoimmune diseases, allergic and inflammatory disorders, allograft rejection, and lymphoid malignancies. Since acute and chronic GC-induced thymic atrophy are associated with increased frequency and severity of infectious diseases [15]–[17], exposure to endogenous or exogenous GCs is arguably the most common type of acquired immunodeficiency in human.

IL-21 is the most recently identified member of the common γ-chain family of cytokines [18]. Produced mainly by activated CD4 T cells, IL-21 was found to: i) promote CD4 T-cell differentiation down the Th17 pathway, ii) co-stimulate activated NK and CD8 lymphocytes, iii) desensitize responding cells to the inhibitory effects of regulatory T cells, and iv) act as a switch for IgG production in B cells [19]. Although IL-21 is not required for hematopoiesis, bone marrow progenitors expand in response to IL-21 overexpression [20], [21]. Likewise, it has been assumed that IL-21 was not essential for thymopoiesis since in IL-21R^−/−^ mice display normal thymic cellularity [21]. However, we recently reported that TCR-engagement during positive selection upregulates IL-21R on the cell surface of DP thymocytes [22]. In contrast to other γc cytokines such as IL-4 or IL-7, IL-21 does not trigger differentiation DP thymocytes to CD8 SP T cells [22]. Instead, IL-21 leads to the expansion of positively selected DP thymocytes. When combined with the differentiation-inducing cytokine IL-7, rIL-21 increases by a 2–3 fold the production of CD8 SP T cells *in* *vitro* [22]. Furthermore, rIL-21 injection to aged mice significantly enhances *de novo* intrathymic T-cell development [22]. To the best of our knowledge, the ability of IL-21 to expand DP thymocytes is unique. Given the mitogenic effect of IL-21 on DP thymocytes, the thymocyte subset which is depleted by GCs, we therefore wished to evaluate whether rIL-21 could enhance recovery from GC-induced thymic atrophy.

## Materials and Methods

### Mice

C57BL/6 female mice (4–6 weeks old) were purchased from the Jackson Laboratory (Bar Harbor, ME). All mice were housed under specific pathogen-free conditions and all experimental protocols were approved by the Comité de Déontologie de l’Expérimentation sur des Animaux from the Université de Montréal (protocol number: 12-045).

### Reagents and Antibodies

The rIL-21 was purchased from Peprotech (Rocky Hill, NJ, USA). All antibodies used in flow-cytometry, and the Cytofix/Cytoperm Kit for intracellular staining were purchased from BD Pharmingen (San Diego, CA, USA). The mouse Vβ TCR screening panel contains 15 pre-diluted FITC-conjugated monoclonal antibodies which recognize mouse Vβ 2, 3, 4, 5.1/5.2, 6, 7, 8.1/8.2, 8.3, 9, 10b, 11, 12, 13, 14, and 17a TCRs.

### Induction of Acute Thymic Atrophy and rIL-21 Administration

To induce acute thymic atrophy, two groups of mice were intraperitoneally (IP)-injected with 1 ug/ml DEX to deplete DP thymocytes. Two days later, DEX-treated mice were IP-injected with rIL-21 (12.5, 25 or 50 ug/kg of weight) in 200 ul PBS on a daily interval (total of three injections). The second DEX-treated group received equal volumes of PBS. One day following the last rIL-21 injection, treated animals were sacrificed and their thymi removed for further analyses. WT C57BL/6 mice injected with PBS only were used as controls for comparative purposes.

### Histology

Thymi were harvested from treated WT C57BL/6 mice, fixed in 10% formalin before being mounted in paraffin. Sections were then stained with hematoxylin and eosin then scanned using the NanoZoomer Digital Pathology system and NPD.scan 2.3.4 software (Hamamatsu) as previously described [23].

### Flow Cytometry Analyses of TECs

Enrichment of thymic stromal cells was performed as described [23], [24]. TECs were defined as CD45^−^CD326^+^ stromal cells.

### qPCR Studies

Thymocytes from treated mice were sorted directly in eppendorf tubes containing 900 µl Trizol (10^6^ cells were collected per tube). Cells were then lysed and the RNA extracted in Trizol reagent. A further RNA purification step was carried out using the RNA extraction kit (QIAgen). Reverse transcription was carried out using the High Capacity cDNA reverse transcription kit and qPCR was performed with a 7900 HT Fast Real-Time PCR system at IRIC’s genomic core facility. Target gene values were normalized to endogenous control *Gapdh*.

### Intracellular Staining

For analysis of pSTAT1, pSTAT3, and pSTAT5, sorted thymocytes were co-cultured in the absence or presence of 10 ng/ml of rIL-21 for 10 min. Cells were then washed prior to intracellular staining by flow-cytometry. Intracellular staining for Bcl2, Bcl2l1 and Bcl6 was performed using PE-labelled primary antibodies according to manufacturer’s instructions.

### OP9-DL1 Cultures

For thymocyte differentiation *in vitro*, sorted DN thymocytes were co-cultured with OP9-DL1 (a generous gift from Dr. *Zúñiga*-*Pflücker*, Sunnybrook Health Sciences Centre, Toronto, Canada) supplemented with rIL-21 or equal volume of PBS for 3, 5 or 7 days [25]. Thymocytes in suspension were then collected, washed with PBS then stained for flow-cytometry analysis.

### Statistical Analyses

*P*-values were calculated using the paired Student *t*-test or ANOVA according to the experimental set-up.

## Results

### Characterization of Thymic IL-21R Expression

**Figure 1:**
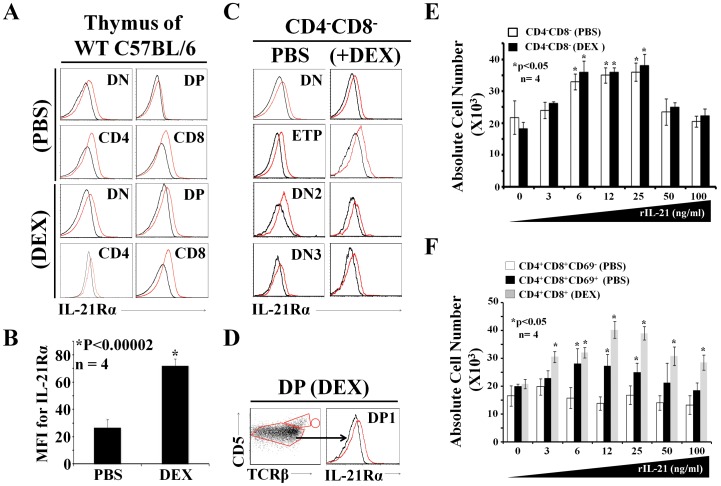
Expression of IL-21Rα and response to IL-21. A) Representative flow-cytometry analysis of IL-21Rα-chain on PBS- (upper panels) versus DEX-treated (lower panels) thymocytes. Isotype control are depicted in black whereas test conditions are displayed in red. B) Numbers indicate mean fluorescent intensity of IL-21Rα-chain expression on the surface of DP thymocytes. We tested 4 mice per group, *P<0.00002. C) Representative flow-cytometry analysis of IL-21Rα-chain on fractionated DN thymocytes from PBS- or DEX-treated mice. The isotype control is shown in black. D) Left panel: distribution of DP1 (TCR^lo^CD5^lo^), DP2 (TCR^int^CD5^hi^) and DP3 (TCR^hi^CD5^int^) subsets among DP thymocytes from DEX-treated mice. Right panel: representative flow-cytometry analysis of IL-21Rα-chain on DP1 thymocytes derived from DEX-treated animals. E–F) Number of cells recovered following *in vitro* culture of the following cell subsets with graded concentrations of IL-21: DN thymocytes derived from PBS- or DEX-treated mice, CD69^−^ and CD69^+^ DP thymocytes from PBS-treated mice, and DP thymocytes derived from DEX-treated mice. We tested 4 mice per group, *P<0.05. All experiments were repeated at least 3 times.

To induce acute thymic atrophy, we used the synthetic GC dexamethasone (DEX), as a single injection of this compound depletes 95% of DP thymocytes within 48 hrs [26]. We then compared IL-21R expression on all thymic populations in untreated and GC-treated mice. According to flow-cytometry analysis, IL-21R is constitutively expressed on the surface of DN and SP thymocytes obtained from C57BL/6 mice injected with PBS or DEX (**Fig. 1A**). Analyses conducted on fractionated DN thymocytes showed similar IL-21R expression on early thymic progenitors (ETPs - cKit^+^CD8β^-^Lin^-^TCRβ^-^CD25^−^), DN2 (cKit^+^CD8β^-^Lin^-^TCRβ^-^CD25^+^) as well as DN3 thymocytes (CD4^−^8^−^Lin^-^CD25^+^CD44^−^) (**Fig. 1C**). While IL-21R was undetectable on the whole DP population from PBS-injected mice, it was clearly upregulated on DP thymocytes from DEX-treated mice (**Fig. 1A–B**). The DP compartment of DEX-treated mice contained almost only DP1 thymocytes (TCRβ^lo^CD5^lo^) with a few DP2 (TCR^int^CD5^hi^) but no DP3 (TCR^hi^CD5^int^) thymocytes (**Fig. 1D**).

To evaluate the functional relevance of these observations, we conducted a series of proliferation assays using thymocytes derived from PBS- or DEX-treated mice cultured *in vitro* with increasing doses of rIL-21 (concentrations ranged from 0–100 ng/ml). DN thymocytes isolated from either group proliferated similarly in a dose-dependent manner as shown by increased absolute cell counts at rIL-21 doses spanning 6–25 ng/ml (**Fig. 1E**). As we previously reported IL-21R expression on the small subset of CD69^+^ (positively selected) DP thymocytes [22], we next compared the proliferative response of three population of DP thymocytes: CD69^+^ and CD69^−^ DP thymocytes from PBS-treated mice and DP thymocytes (all CD69^−^) from DEX-treated mice. In PBS-treated mice, only 6% of DP thymocytes were CD69^+^ and all DP thymocytes from DEX-treated mice were CD69^−^. CD69^−^ DP thymocytes from PBS-treated mice were unresponsive to rIL-21 (**Fig. 1F**). Both CD69^+^ DP thymocytes from the PBS group and DP thymocytes from the DEX group expanded in the presence of rIL-21. However, their dose-response ranges differed (6–25 ng/ml for CD69^+^ DP thymocytes versus 3–100 ng/ml for DEX-derived DP thymocytes) (**Fig. 1F**). Hence, the salient finding here is that DEX treatment upregulates IL-21R on DP thymocytes and render them responsive to IL-21. While IL-21R is normally found in a tiny proportion of DP thymocytes (CD69^+^) it is found on a large fraction of (CD69^−^) DP thymocytes following DEX treatment.

### Administration of rIL-21 Accelerates Thymic Recovery following DEX-mediated Acute Atrophy

Since DEX modulates IL-21R expression on DP thymocytes, we next investigated the impact of *in vivo* rIL-21 administration on thymus function following induction of acute thymic atrophy. In order to optimize the recovery response, we tested three rIL-21 doses (12.5, 25 or 50 ug/kg of weight) injected on a daily basis (total of three injections) versus equivalent volumes of PBS, starting on day 2 after DEX administration (on day 0). On day 5, animals were sacrificed for analysis of thymic tissues. No differences were found in size, weight or cellularity of thymi from animals injected with low (12.5 ug/kg) or high (50 ug/k) doses of rIL-21 (**Fig. S1 and S2** **respectively**). Notably, the highest rIL-21 dose (50 ug/kg) triggered a major accumulation of DN thymocytes as this population represented 77% of thymocytes as opposed to 13% in DEX/PBS group or 3% in PBS control mice (**Fig. S2**). In depth flow-cytometry fractionation analysis of the DN population derived from animals injected with the highest rIL-21 dose revealed a blockade at the DN1 stage with a significant increase in the frequency of ETPs which reached 1.6% as opposed to 0.3–0.5% in control mice (**Fig. S3**). To verify whether such blockade was reversible *in vitro*, we co-cultured DN thymocytes derived from mice treated with 50 ug/kg rIL-21 with OP9-DL1 and followed their differentiation to the DP stage at three time points over 7 days. When DN thymocytes derived from high dose animals were co-cultured with OP9-DL1 cells without further rIL-21 supplementation *in vitro* (PBS control), most of them differentiated to the DP stage (**Fig. S4**). Interestingly however, sustained *in vitro* supplementation of high dose rIL-21 (100 ng/ml) severely blocked DN differentiation to the DP stage. These data suggest that high doses of rIL-21 induce a reversible blockade in the differentiation of DN thymocytes (**Fig. S4**).

**Figure 2:**
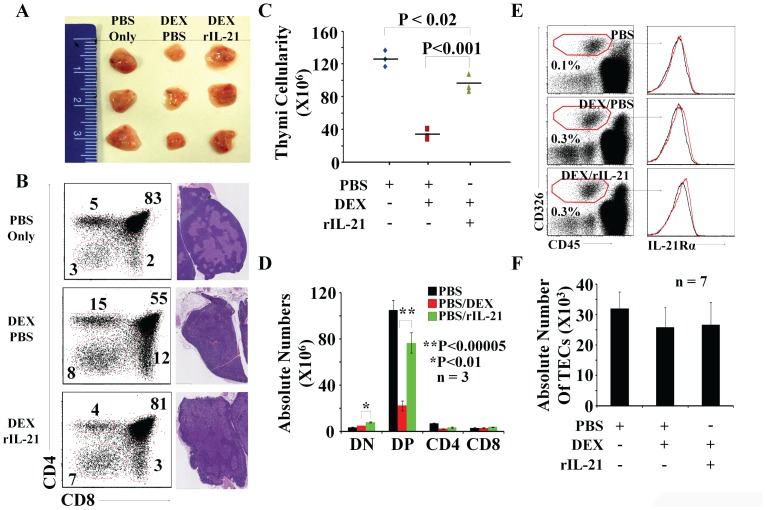
The effect of rIL-21 administration on thymic recovery following DEX treatment. A) A representative photograph of thymi derived from treated mice. A group of C57BL/6 mice received equivalent volume of PBS. B) Flow-cytometry/histological analysis of thymi derived from treated mice. C) Total thymic cellularity for all tested groups. D) Absolute numbers of DN, DP, CD4 and CD8 SP T cells from thymi derived from treated mice. PBS only (black), DEX/PBS (red) and DEX/rIL-21 (green). We tested 3 mice per group. Data are representative of 3 separate experiments. E) Representative flow-cytometry analysis of TECs derived from PBS-, DEX/PBS- versus DEX/rIL-21-treated mice. Following TECs gating, IL-21Rα-chain was assessed by flow-cytometry. Isotype controls are displayed by black histogram whereas test condition is represented by red histograms. F) Absolute numbers of TECs from thymi derived from treated mice. We tested 7 mice per group. Data are representative of 3 separate experiments.

However, the key observation here is that administration of rIL-21 at an intermediate dose of 25 ug/kg of weight significantly increased thymic size in comparison to DEX-PBS-control mice (**Fig. 2A**). Increased thymic cellularity in this group involved mainly DP thymocytes whose frequency increased from 55% in the PBS group to 81% in recipients of rIL-21 (25 ug/kg of weight) (**Fig. 2B** **- left panels**). Overall thymic architecture, and cortico-medullary demarcation in particular, were better preserved in recipients of intermediate doses of IL-21 than in DEX-PBS treated mice (**Fig. 2B** **- right panels**). Moreover, rIL-21 administration to DEX-treated mice enhanced overall recovery of thymic cellularity (**Fig. 2C**) with a significant increase in absolute number of DN and DP thymocytes (**Fig. 2D**). TECs express GC receptors [27], [28] and might therefore be affected by GCs. This led us to investigate whether, in addition to its effect on thymocytes, IL-21 might also affect TECs. We found that TECs were negative for IL-21R expression even after DEX administration (**Fig. 2E**). Furthermore, TEC absolute numbers remained stable under all tested conditions (**Fig. 2F**). We conclude that intermediate doses of rIL-21 accelerate thymic recovery from GC-induced atrophy via thymocyte-intrinsic TEC-independent mechanism(s).

### Administration of rIL-21 Upregulates *Bcl6* in DP Thymocytes

**Figure 3:**
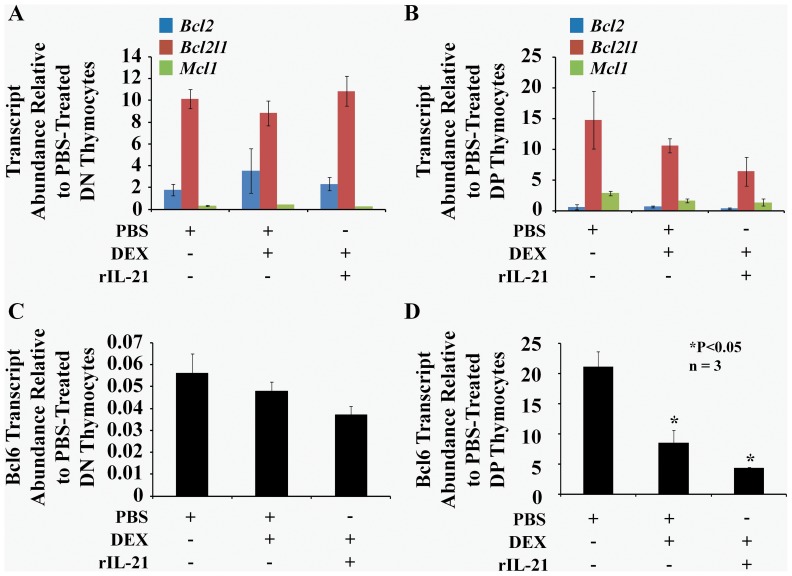
Transcript analysis of molecules implicated in thymocyte survival and expansion. A–B) qPCR analysis of *Bcl2*, *Bcl2l1* and *Mcl1* or (C–D) *Bcl6* expression in freshly harvested DN (A–C) or DP (B–D) thymocytes derived from PBS-, DEX/PBS- or DEX/rIL-21-treated WT C57BL/6 mice. Data depict transcript abundance relative to DN (A–C) or DP (B–D) thymocytes. We tested 3 mice per group, *P<0.05. All experiments have been repeated 3 times.

**Figure 4:**
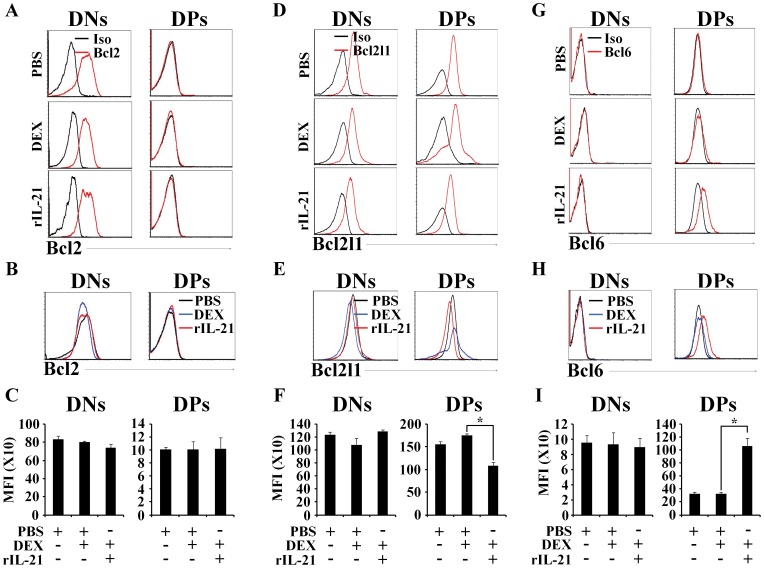
Administration of rIL-21 to DEX-injected animals induces Bcl-6 expression in DP thymocytes. A–B) Representative flow-cytometry analysis of Bcl2 expression in DN and DP thymocytes derived from PBS-, DEX/PBS- or DEX/rIL-21-treated mice. C) Compiled mean fluorescent intensity for Bcl2 analysis. D–E) Representative flow-cytometry analysis for Bcl2l1 in DN and DP thymocytes from the same experimental groups. F) Compiled mean fluorescent intensity for Bcl2l1 analysis. G–H) Representative flow-cytometry analysis for Bcl6 in DN and DP thymocytes using same experimental groups. I) Compiled mean fluorescent intensity for Bcl6 analysis. For all experiments performed, we tested 3 mice per group, *P<0.05. Data shown are representative of 3 separate experiments.

Stress-induced thymic atrophy has been shown to be associated with several intrathymic alterations in gene expression [8], [29], [30]. Many of these changes involve genes that can impinge on cell cycle progression, cell survival and cell maintenance. IL-21 drives the expression of several genes involved in proliferation, survival and cell differentiation [18]. We therefore sought to determine whether the beneficial effect of IL-21 on thymopoiesis following exposure to GCs correlated with changes in expression of genes known to regulate thymocyte survival or proliferation: *Bcl2, Bcl2l1, Mcl1, or Bcl6* [31], [32]. We found no changes in expression of *Bcl2, Bcl2l1* or *Mcl1* in DN or DP thymocytes from PBS-, DEX/PBS- or DEX/rIL-21-treated mice (**Fig. 3A**). Notably, expression of *Bcl6* transcripts was significantly decreased in DP thymocytes following DEX/PBS-treatments and was further decreased upon rIL-21 administration (**Fig. 3B**). We next evaluated by intracellular staining the expression of each gene at the protein level (except for Mcl-1 due to the absence of commercially available antibodies) in thymocytes from PBS-, DEX/PBS- or DEX/rIL-21-treated mice. Bcl2 was undetectable in DP thymocytes and its expression was invariant in DN thymocytes from the three experimental groups as depicted by histogram overlap (**Fig. 4A–B**) and compiled mean fluorescent intensities (MFIs) (**Fig. 4C**). Expression of Bcl2l1 was unaffected in DN thymocytes (**Fig. 4D–F**), but was decreased in DP thymocytes from the DEX/rIL-21 group (**Fig. 4F**). Finally, levels of Bcl6 were invariant in DN thymocytes but were upregulated by 5-fold in the group treated with rIL-21 (**Fig. 4G–I**). We conclude that rIL-21 administration led to a strong upregulation of Bcl6 in DP thymocytes. Bcl6 expression is regulated at the transcriptional and post-transcriptional levels [33]. The discrepancy between mRNA and protein levels means that IL-21-induced upregulation of Bcl6 in DP thymocytes is due to post-trancriptional mechanisms (e.g., protein degradation by the ubiquitin-proteasome system). The function of Bcl6 has been particularly well characterized in germinal center B cells where Bcl6 acts as a transcriptional repressor that prevents apoptosis and enhances tolerance of genomic breaks [33]. Since DP thymocytes undergo genomic breaks and display a high apoptosis rate [34], upregulation of Bcl6 can well explain the expansion of DP thymocytes induced by IL-21.

### Analysis of STAT Phosphorylation Responses following *in vitro* rIL-21 Stimulation

**Figure 5:**
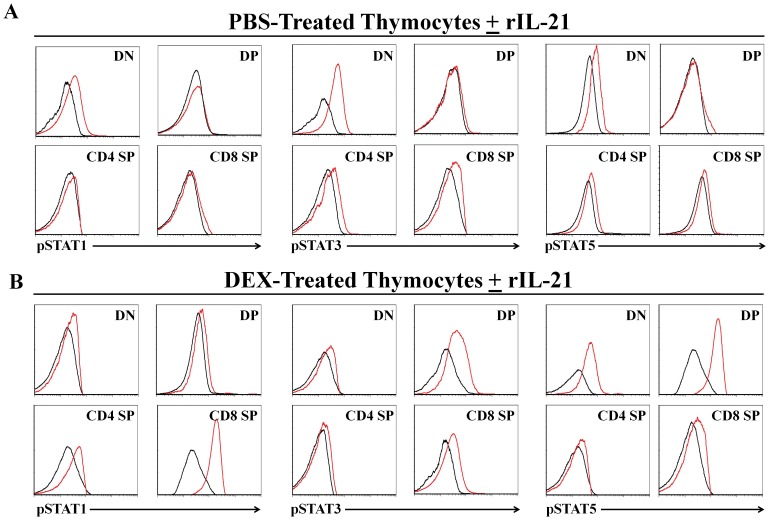
STAT phosphorylation in thymocytes. A–B) Representative flow-cytometry analysis of pSTATs in fractionated thymocytes derived from PBS- or DEX-treated animals supplemented with 10ng/ml rIL-21 (red histograms) or no cytokines (black histograms). We tested 3–6 mice per group in separate experiments.

The receptor for each γc cytokine, including IL-21, activates JAK1 and JAK3, and the signals are transduced by STAT proteins [35]. Given that expression of the IL-21R is induced on the surface of DP thymocytes following DEX treatment (**Fig. 1A–B**), we presumed that rIL-21 stimulation may lead to differential STAT signaling pattern in DP thymocytes derived from PBS- versus DEX-injected mice. We therefore evaluated the effect of *in vitro* rIL-21 supplementation on thymocytes harvested from PBS- versus DEX-injected mice. In the PBS group, rIL-21 increased the levels of phospho-(p)STAT1 in DN thymocytes (**Fig. 5A left panel** **s**), and of pSTAT3 and pSTAT5 in both DN and SP thymocytes (**Fig. 5A** **middle).** As expected, no phosphorylation of tested STAT molecules was detected in DP thymocytes from PBS-injected mice (**Fig. 5A right panel** **s**). Responses to rIL-21 were different in thymocytes from DEX-treated mice relative to thymocytes from PBS-injected mice. The salient observation was that in DEX-treated mice, rIL-21 led to a mild increase in levels of pSTAT1 and major increases in pSTAT3 and pSTAT5 (**Fig. 5B**). Besides, relative to the PBS group, thymocytes from DEX-treated mice displayed more important upregulation of pSTAT1 in CD8 SP thymocytes and of pSTAT5 in DN thymocytes (**Fig. 5**). We conclude that DEX-induced responsiveness of DP thymocytes to IL-21 leads to phosphorylation of STAT3, STAT5, and to a lesser extent, STAT1.

### Characterization of TCRVβ-chains Expression on SP T Cells

**Figure 6:**
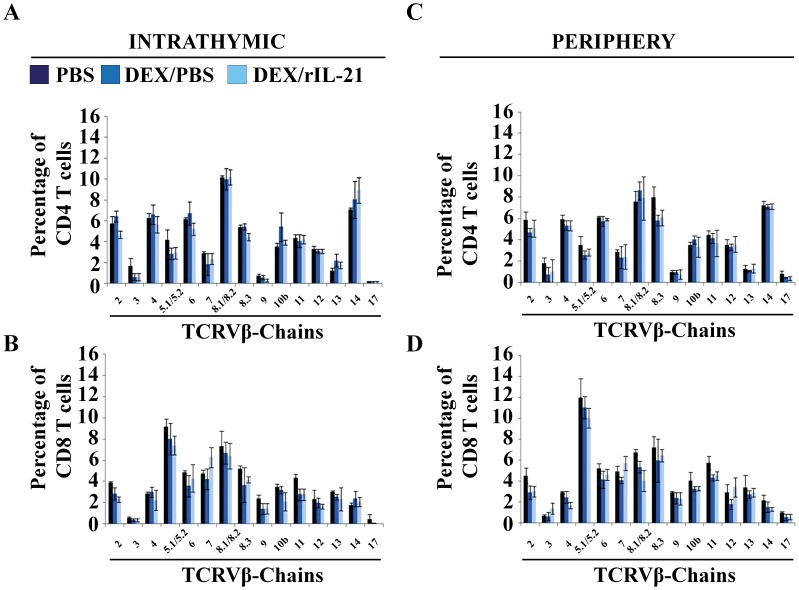
Analysis of TCR diversity. A–B) Flow-cytometry analysis of 15 TCRVβ-chains using intrathymic CD4 (A) or CD8 (B) SP T cells. C–D) Similar flow-cytometry analyses were performed using spleen CD4 (A) or CD8 (B) SP T cells. For both analyses, T cells were derived from PBS- (dark blue), DEX/PBS- (blue) or DEX/rIL-21-treated mice (light blue). We tested 3 mice per group. Data shown are representative of 3 separate experiments.

Considering the altered signaling pattern observed in the DP fraction following treatment with DEX, we sought to determine whether rIL-21 administration under such circumstances might introduce a bias in the TCR repertoire. To address this possibility, we analyzed the expression of 15 TCRVβ-chains on the surface of CD4 and CD8 SP T cells derived from PBS only-, DEX/PBS- or DEX/rIL-21-treated mice. No perturbation of the TCRVβ repertoire diversity was found in intrathymic (**Fig. 6C–D**) or peripheral (splenic) CD4 and CD8 SP T-cell populations (**Fig. 6C–D**). We conclude that rIL-21-induced recovery from GC-induced thymic atrophy does not cause a skewing of the TCRVβ repertoire.

## Discussion

**Figure 7:**
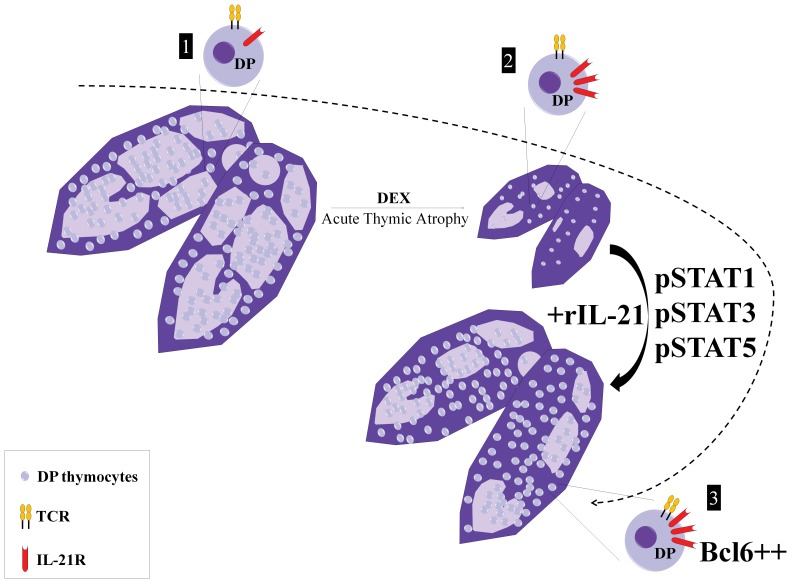
Proposed model for rIL-21- accelerated recovery following induction of acute thymic atrophy. Based on data presented herein, we propose the following model: under normal circumstances, only a small fraction of DP thymocytes (CD69^+^ post-selection DP3s which represent 6% of DPs) express the IL-21R (step 1). Upon DEX administration, 90–95% of DP thymocytes are depleted by apoptosis within 48 hrs (step 2). At that stage, the thymic cortico-medullary demarcation is blurred. The surviving DPs (CD69^−^ DP1s) upregulate IL-21R on their cell surface and become responsive to rIL-21. Upon rIL-21 administration, pSTAT1, pSTAT3 and pSTAT5 are activated while post-transcriptional mechanisms lead to accumulation of Bcl6. These signaling events accelerate thymic recovery from DEX-induced atrophy.

In addition to caspase activation and induced cell death of DP thymocytes, supraphysiological levels of GCs inhibit thymopoiesis by disrupting the expression of several molecules forming the TCR complex [29], [30], [36]–[40]. Furthermore, significant changes to the cytoskeleton and extracellular matrix occur in thymi exposed to high levels of GCs as evidenced by loss of the cortico-medullary demarcation [8]. Herein, we report that DEX treatment triggers the expression of the IL-21R on surviving DP thymocytes. As a result, administration of rIL-21 to DEX-treated mice led to pSTAT1, pSTAT3 and pSTAT5 activation as well as accumulation of Bcl6 via post-transcriptional mechanisms. Thereby, IL-21 substantially accelerated thymic recovery from GC-induced atrophy (**Fig. 7**).

The thymus is mainly composed of T-cell progenitors and a minor TEC population necessary for supporting thymocyte development [24]. As TECs constitutively express GC receptors, DEX administration at high-dose (20 mg/Kg) has been reported to degenerate the thymic epithelium through TECs-induced differentiation to adipocytes [27]. Although we did not directly assess such trans-differentiation or expression of pre-adipocytic markers, no significant changes in TEC population numbers were observed in our DEX model. The apparent discrepancy between our observations and those reported by Talaber and colleagues may be due to the low dose of DEX used in our experiments (5 mg/kg).

We anticipated rIL-21-mediated enhancement of thymic recovery to be driven by thymocyte expansion as well as increased survival via induction of anti-apoptotic molecules. Expression profiling of Bcl2, Bcl2l1 and Mcl1 at the transcript and protein levels clearly discarded their involvement in the recovery process as their expression was either diminished (for Bcl2l1) or not modulated by rIL-21 (**Fig.** **3**–**4**). Interestingly however, Bcl6 protein (but not mRNA) levels were upregulated by 5-fold in DP thymocytes following rIL-21 administration (**Fig. 4**). This observation is consistent with our previous findings showing that *in vitro* rIL-21 treatment of post-selected DP thymocytes up-regulate Bcl6 expression [22]. Expression of the *Bcl6* gene is tightly regulated through auto-regulatory loop in which the Bcl6 protein binds to the regulatory region of its own gene limiting therefore its expression [41]–[43]. Consistent with this notion, the down-regulation in *Bcl6* expression observed by qPCR within the DP fraction derived from GC-treated mice correlated with increased Bcl6 protein levels. Under physiological conditions, *Bcl6* is known to be mainly expressed in germinal center B cells [33], [41]–[43]. Following antigen activation, B cells undergo somatic hypermutation and class switch recombination to improve immunoglobulin affinity [33], [44]. Such processes involve DNA breaks which may heavily affect the survival of germinal center B cells. Interestingly, Bcl6 has been shown to play a central role in sustaining tolerance to DNA damage thus preventing premature B-cell death [33]. This function is particularly interesting as DP thymocytes development culminates in TCR rearrangements akin to B-cell receptor-specific somatic hypermutation. Therefore, Bcl6 induction in DP thymocytes following rIL-21 administration to GC-treated mice may suggest a role played by this transcriptional repressor in promoting the proliferation of GC-stressed DP thymocytes while preserving tolerance to genomic damages taking place during positive selection.

Nonetheless, a number of signaling pathways involved in the response to chemokines and cytokines are modulated by Bcl6 [33]. In macrophages for instance, Bcl6 dampens inflammatory signaling through repression of chemokine expression and target genes of the transcription factor NF-κB [45], [46]. Notably, *Bcl6^−/−^* mice develop a lethal inflammatory disease caused by the interaction and crosstalk between macrophages and helper T cells [47]. Bcl6 has been also reported to bind and repress the expression of a number of target genes in B cells [45]. These include the B-cell differentiation factor (Blimp1), cell cycle regulators (cyclin D2, p27*kip1*), lymphocyte activation genes (*CD44*, *CD69*), and chemokines (macrophage-inflammatory protein 1, interferon-inducible protein-10) [45], [48]. Although we do not know the identity of target genes regulated by Bcl6 in T cells, studies performed in B cells and macrophages suggest that Bcl6 is involved in suppressing inflammatory responses, which are known to exacerbate thymic atrophy [8]. Future studies to be conducted using *Bcl6* ^−/−^ mice might be necessary to dissect the precise molecular mechanism by which this protein controls intrathymic inflammation, expansion and perhaps T-cell progenitors differentiation.

Nowadays, IL-21 is exploited in various clinical trials as a cancer immunotherapeutic agent [49], [50] or for the control of persistent viral infections such as HIV [51]. Based on compiled clinical data, rIL-21 administration is relatively safe with minor side effects such as of flu-like symptoms. We therefore infer that rIL-21 holds the potential to stimulate thymic functions while limiting perhaps tissue destruction during inflammatory responses induced by harmful stress conditions. Taken together, our data may pave the way to the clinical use of rIL-21 for enhancing thymopoiesis during acute stress-induced atrophy prior to vaccination or for T-cell reconstitution following bone marrow transplantation.

## Supporting Information

Figure S1 **The effect of low dose rIL-21 administration on thymic recovery following DEX treatment.** A) Schematic diagram of the experimental outline. B) A representative photograph of thymi derived from treated mice. A group of WT C57BL/6 mice received equivalent volume of PBS. C–D) Thymic weight and cellularity respectively. E) Flow-cytometry analysis of thymi derived from PBS, DEX/PBS, or DEX/rIL-21 groups. F) Absolute number of thymic subsets derived for all tested groups. We tested 3 mice per group. Data are representative of 3 separate experiments.
(PDF)

Figure S2 **The effect of high dose rIL-21 administration on thymic recovery following DEX treatment.** A) Schematic diagram of the experimental outline. B) A representative photograph of thymi derived from PBS versus rIL-21 injection of DEX-treated mice. A group of WT C57BL/6 mice received equivalent volume of PBS. C–D) Thymic weight and cellularity respectively. E) Flow-cytometry analysis of thymi derived from PBS, DEX/PBS, or DEX/rIL-21 groups. F) Absolute number of thymic subsets derived for all tested groups. We tested 3 mice per group. Data are representative of 3 separate experiments.
(PDF)

Figure S3 **Gating strategy for the analysis of ETP, DN2, DN3 and DN4 progenitors in mice injected with high dose rIL-21.** A) Representative flow-cytometry analysis for ETP and DN2 gating. B) ETP and DN2 percentages obtained using same gating strategy in (A). C) Representative flow-cytometry analysis for DN3 and DN4 gating. D–E) Total DN1 (D) or DN3/DN4 (E) percentages obtained using same gating strategy in (C). We tested 3 mice per group, *P<0.05. Data shown are representative of 3 separate experiments.
(PDF)

Figure S4 **DN thymocytes differentiation to DP thymocytes.** A) Representative flow-cytometry analysis of DN thymocytes co-cultured on OP9-DL1. DN thymocytes were derived from DEX-treated animals injected with PBS, 25 ug/kg or 50 ug/kg of rIL-21. They were then co-cultured on OP9-DL1 for 3, 5 or 7 days in the presence of PBS, 10 ng/ml rIL-21 or 100 ng/ml rIL-21. B) Percentages of in vitro differentiated DP thymocytes using the same DN thymocytes listed in (A). We tested 3 mice per group. Data are representative of 3 separate experiments.
(PDF)
