# Supplementary material for: Interleukin-21 Accelerates Thymic Recovery from Glucocorticoïd-Induced Atrophy
Source: PLoS One. 2013 Sep 2;8(9):e72801. doi: 10.1371/journal.pone.0072801 (PMC3759406; doi:10.1371/journal.pone.0072801)
Supplement: Figure S1 — The effect of low dose rIL-21 administration on thymic recovery following DEX treatment. A) Schematic diagram of the experimental outline. B) A representative photograph of thymi derived from treated mice. A group of WT C57BL/6 mice received equivalent volume of PBS. C–D) Thymic weight and cellularity respectively. E) Flow-cytometry analysis of thymi derived from PBS, DEX/PBS, or DEX/rIL-21 groups. F) Absolute number of thymic subsets derived for all tested groups. We tested 3 mice per group. Data are representative of 3 separate experiments. (PDF) [file pone.0072801.s001.pdf]

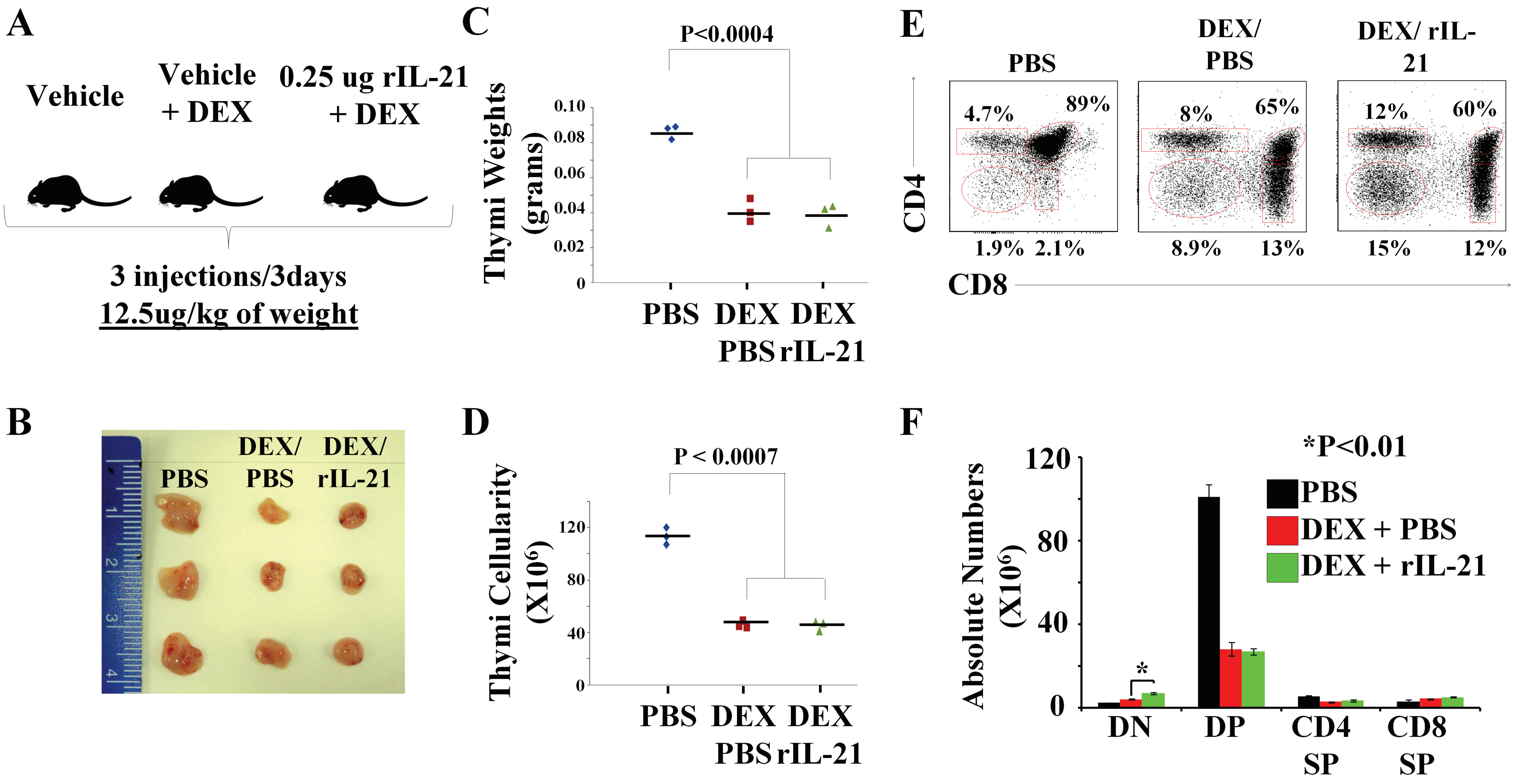

Figure S1. The effect of low dose rIL-21 administration on thymic recovery following DEX treatment. A) Schematic diagram of the experimental outline. B) A representative photograph of thymi derived from treated mice. A group of WT C57BL/6 mice received equivalent volume of PBS. C-D) Thymic weight and cellularity respectively. E) Flow-cytometry analysis of thymi derived from PBS, DEX/PBS, or DEX/rIL-21 groups. F) Absolute number of thymic subsets derived for all tested groups. We tested 3 mice per group. Data are representative of 3 separate experiments.

FIGURE S1
