# Supplementary material for: Interleukin-21 Accelerates Thymic Recovery from Glucocorticoïd-Induced Atrophy
Source: PLoS One. 2013 Sep 2;8(9):e72801. doi: 10.1371/journal.pone.0072801 (PMC3759406; doi:10.1371/journal.pone.0072801)
Supplement: Figure S3 — Gating strategy for the analysis of ETP, DN2, DN3 and DN4 progenitors in mice injected with high dose rIL-21. A) Representative flow-cytometry analysis for ETP and DN2 gating. B) ETP and DN2 percentages obtained using same gating strategy in (A). C) Representative flow-cytometry analysis for DN3 and DN4 gating. D–E) Total DN1 (D) or DN3/DN4 (E) percentages obtained using same gating strategy in (C). We tested 3 mice per group, *P<0.05. Data shown are representative of 3 separate experiments. (PDF) [file pone.0072801.s003.pdf]

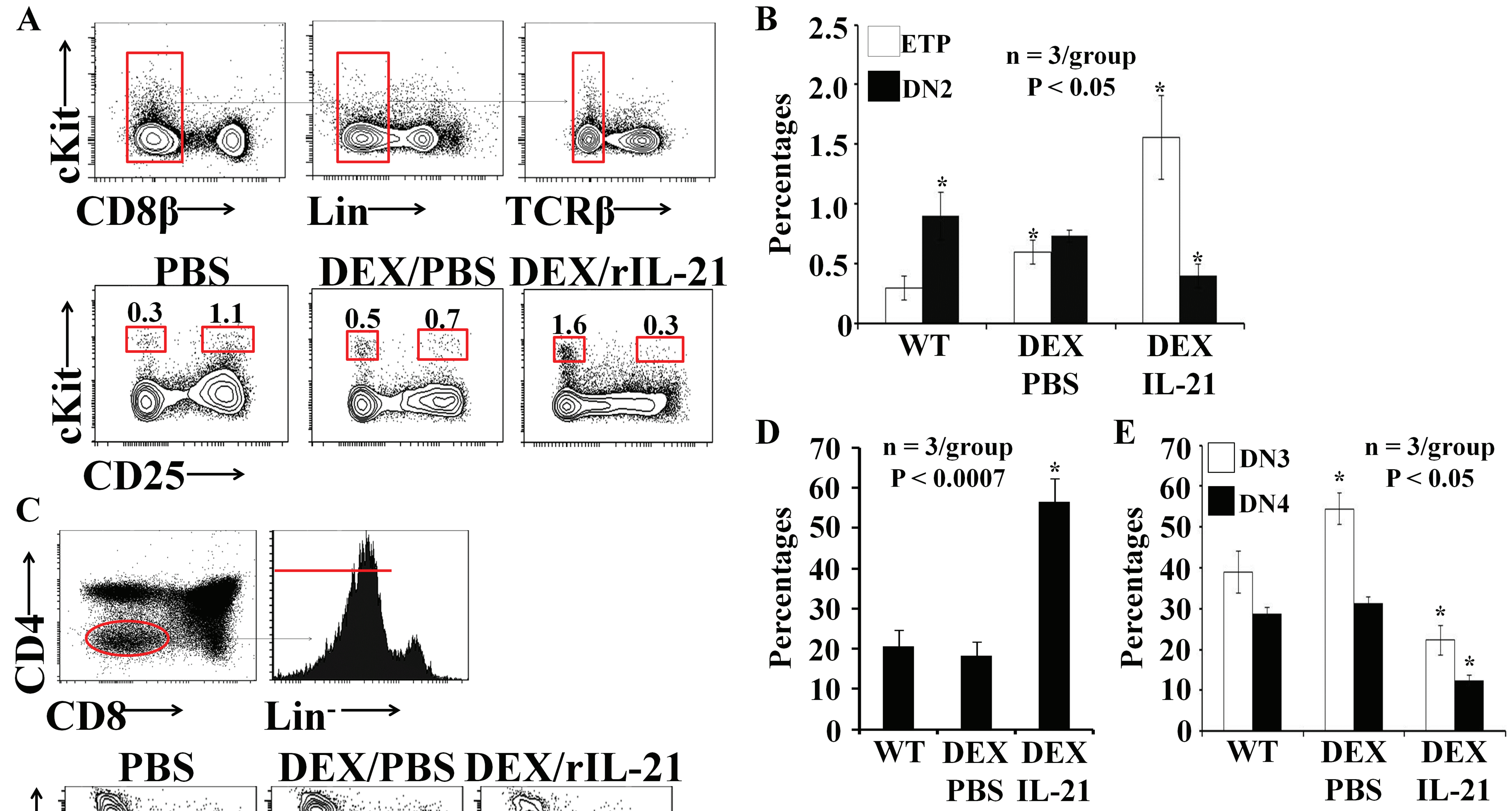
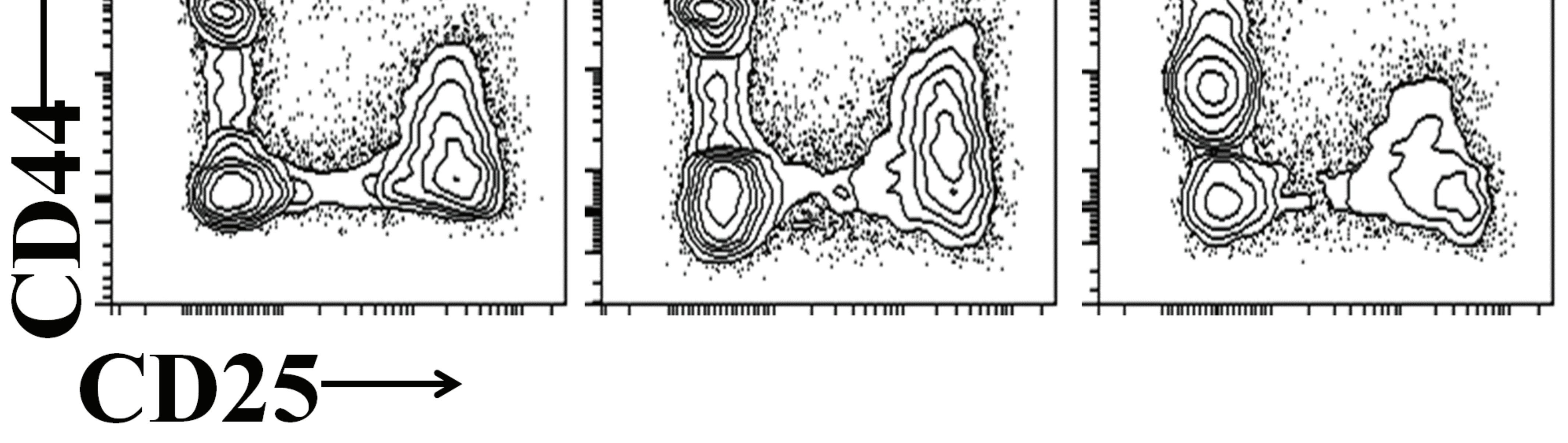

FIGURE S3

Figure S3. Gating strategy for the analysis of ETP, DN2, DN3 and DN4 progenitors in mice injected with high dose rIL-21. A) Representative flow-cytometry analysis for ETP and DN2 gating. B) ETP and DN2 percentages obtained using same gating strategy in (A). C) Representative flow-cytometry analysis for DN3 and DN4 gating. D-E) Total DN1 (D) or DN3/DN4 (E) percentages obtained using same gating strategy in (C). We tested 3 mice per group, *$P < 0.05$. Data shown are representative of 3 separate experiments.
