# Supplementary material for: Interleukin-21 Accelerates Thymic Recovery from Glucocorticoïd-Induced Atrophy
Source: PLoS One. 2013 Sep 2;8(9):e72801. doi: 10.1371/journal.pone.0072801 (PMC3759406; doi:10.1371/journal.pone.0072801)
Supplement: Figure S4 — DN thymocytes differentiation to DP thymocytes. A) Representative flow-cytometry analysis of DN thymocytes co-cultured on OP9-DL1. DN thymocytes were derived from DEX-treated animals injected with PBS, 25 ug/kg or 50 ug/kg of rIL-21. They were then co-cultured on OP9-DL1 for 3, 5 or 7 days in the presence of PBS, 10 ng/ml rIL-21 or 100 ng/ml rIL-21. B) Percentages of in vitro differentiated DP thymocytes using the same DN thymocytes listed in (A). We tested 3 mice per group. Data are representative of 3 separate experiments. (PDF) [file pone.0072801.s004.pdf]

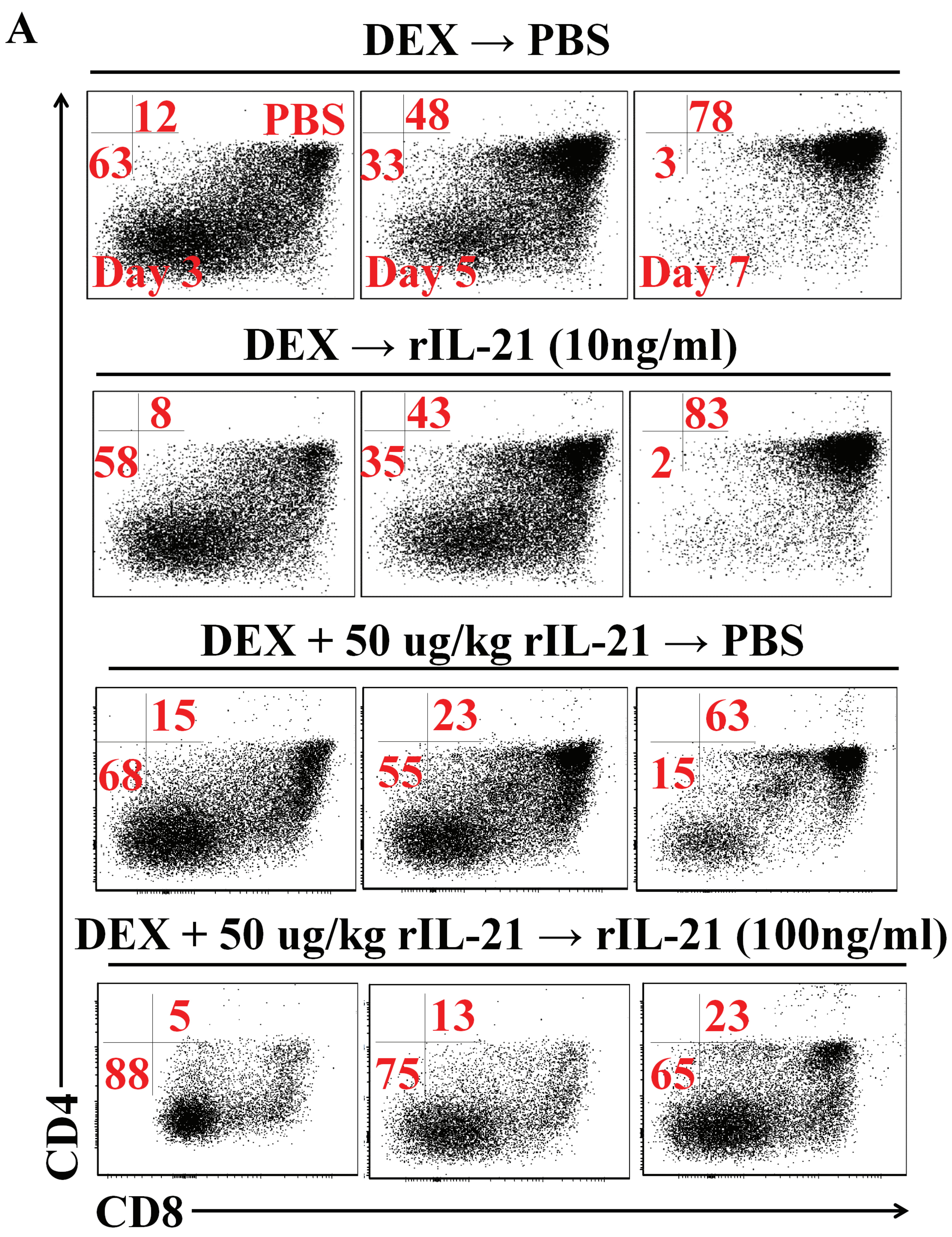

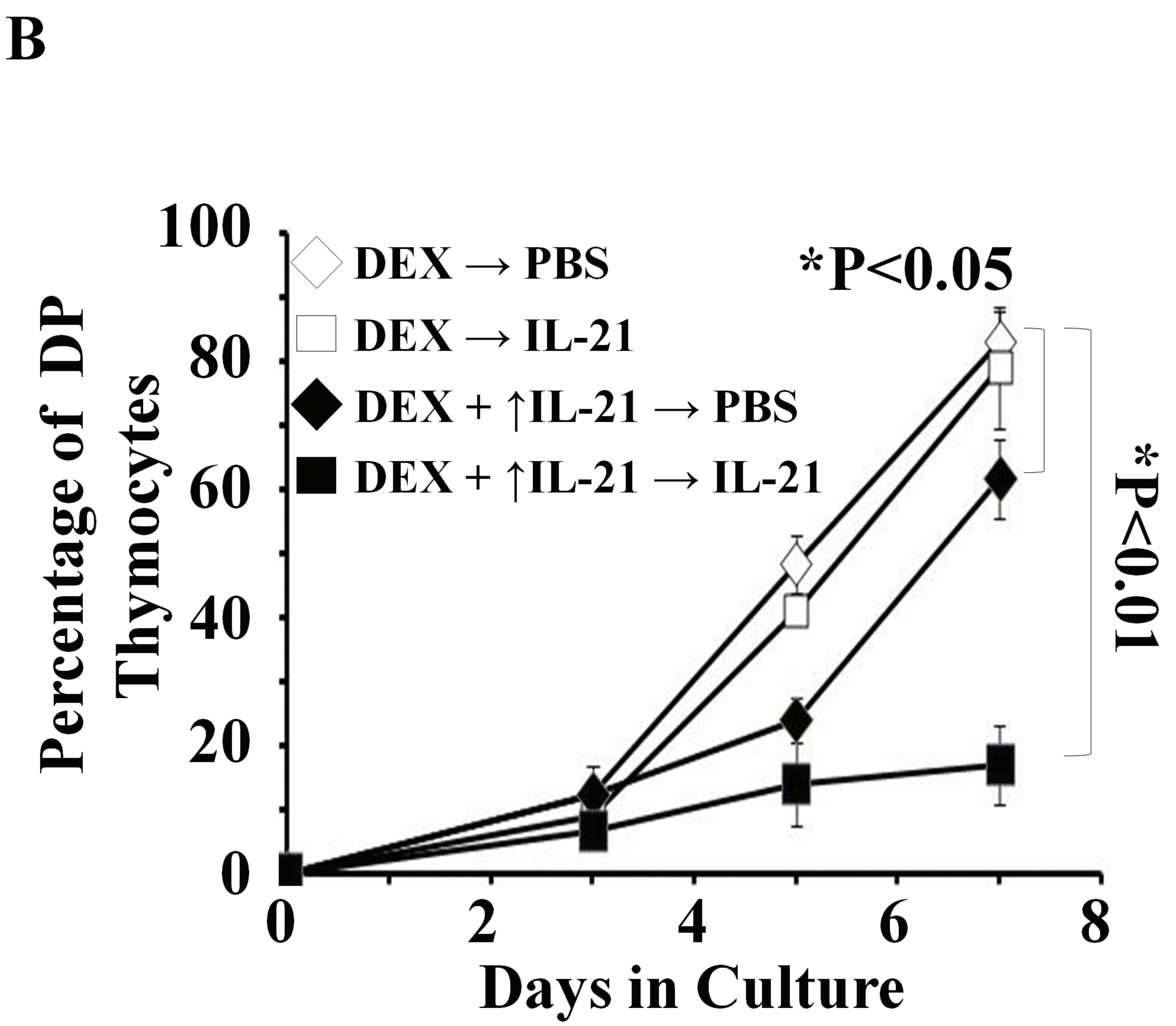

Figure S4. DN thymocytes differentiation to DP thymocytes. A) Representative flow-cytometry analysis of DN thymocytes co-cultured on OP9-DL1. DN thymocytes were derived from DEX-treated animals injected with PBS, 25ug/kg or 50ug/kg of rIL-21. They were then co-cultured on OP9-DL1 for 3, 5 or 7 days in the presence of PBS, 10ng/ml rIL-21 or 100ng/ml rIL-21. B) Percentages of in vitro differentiated DP thymocytes using the same DN thymocytes listed in (A). We tested 3 mice per group. Data are representative of 3 separate experiments.

FIGURE S4
